# A Comprehensive Analysis of the Clinical Significance and Underlying Oncogenic Roles of Specific MMPs in Gastric Carcinoma Reveals their Potential Roles in Prognosis and Therapy

**DOI:** 10.2174/0115665240309837241204184939

**Published:** 2025-01-03

**Authors:** Shiyang Jin, Jing Wang, Kuan Wang

**Affiliations:** 1 Department of Gastrointestinal Surgery, Harbin Medical University Cancer Hospital, Harbin, China;; 2 Department of Breast Cancer Surgery, Harbin Medical University Cancer Hospital, Harbin, China

**Keywords:** Gastric cancer, matrix metalloproteinase, biomarkers, molecular targeted therapy, predictive modeling, MMPs

## Abstract

**Background:**

Gastric cancer is a major global cause of cancer-related deaths, necessitating investigation into Matrix Metalloproteinases’ (MMPs) diagnostic and prognostic value. Our study aimed to analyze their significance in gastric cancer.

**Methods:**

We evaluated MMP family genes' mRNA and protein expression using the University of Alabama at Birmingham (UALCAN) and Human Protein Atlas (HPA) databases. Then, we analyzed the relationship between their mRNA expression and gastric cancer staging and survival using Gene Expression Profiling Interactive Analysis (GEPIA) and Kaplan–Meier plotter. Furthermore, we assessed this family’s gene mutation rates in gastric cancer patients using Search Tool for the Retrieval of Interaction Genes/Proteins (STRING) and explored potential pathways and mechanisms *via* Database for Annotation, Visualization, and Integrated Discovery (DAVID), cBioPortal, and R. Finally, we established a predictive model for gastric cancer based on these analyses to understand these genes’ roles in cancer.

**Results:**

Our findings revealed significantly upregulated mRNA expression of MMP1/2/3/7/9/10/11/12/13/14 in gastric cancer tissues (p<0.05). Higher levels of MMP2/7/10-encoded proteins (middle or high) were observed in tumor tissues, with MMP2/11/14 closely associated with different cancer stages (p<0.05). Additionally, MMP2/7/11/14/20 mRNA levels correlated with short-term overall survival (about 20 months), while MMP1/3/9/12/13 expression was associated with favorable overall survival (about 30 months). Gastric cancer patients exhibited a 21% mutation rate of MMP family genes, which correlated with favorable overall survival. Enrichment analysis and protein-protein interaction results underscored the close association of MMPs with gastric cancer development. The MMP2 model demonstrated a significant decline in survival rates for the high expression group, with a Hazard Ratio (HR) of 1.78 (95% CI 1.47-2.16) and a log-rank P value of 2.9e-09. Statistical significance was set at p < 0.05. Univariate Cox regression identified MMP2 as a risk factor for gastric cancer patients.

**Conclusion:**

Our findings highlighted MMPs' essential role in gastric cancer progression, impacting patient survival. MMP2 emerged as a promising target for gastric carcinoma detection and treatment.

## 1 INTRODUCTION

Gastric cancer ranks fifth in terms of global total mortality among all cancers. Additionally, China has one of the highest global incidences of gastric cancer [1-3]. While the incidence and fatality rates of gastric carcinoma have been consistently decreasing in the last decade, accurate diagnosis and treatment of gastric cancer still pose major challenges to clinicians. Investigating the molecular features of gastric cancer is crucial for identifying possible biomarkers that may serve as prognostic and diagnostic indicators, as well as potential treatment targets [4-7]. However, most compounds with potential prognostic value have not yet been investigated in depth. Matrix Metalloproteinase (MMP) family members play an important role in the function of cells and they have always been one of the major gene families in tumor research [8-11]. An analysis of MMP genes may be useful in the diagnosis and treatment of gastric cancer [12].

Certain zinc-dependent endopeptidases, known as MMPs, regulate the turnover speed of components within the Extracellular Matrix (ECM) [13]. They were discovered by Gross and Lapiere in 1962, after examining tissue samples from the tails of tadpoles. Subtypes of MMPs have been characterized according to their substrate specificity and localization. MMPs regulate several key physiological activities, including tissue remodeling, including angiogenesis, bone development, wound healing, and uterine or mammary involution [14]. They also influence cellular processes, such as cell proliferation, migration, and adhesion [15-17]. Some research studies have explained that MMPs are essential for the initiation, proliferation, and metastasis of carcinoma by breaking down the physical barriers within the ECM [18-20]. MMP overexpression is associated with a worse cancer prognosis. Several studies have observed an increase in the mRNA levels of MMPs in gastric carcinoma cells [21-23]; however, there is still no clear understanding of the role of each member of the MMP family in the growth and advancement of gastric cancer.

Based on the literature, we selected 11 genes encoding MMPs (MMP1/2/3/7/9/10/11/12/13/14/20). These genes have been reported multiple times in previous cancer-related studies [12, 13, 15-23]. In this study, we conducted a thorough analysis to determine the significance of the different MMP family members in gastric cancer. Using multiple public databases, we examined the genetic mutations and mRNA expressions in the gastric cancer samples to determine their prognostic and diagnostic values. The 100 genes that the MMPs co-expressed with were also examined in order to identify the probable pathways and prospective functions of the MMPs. At last, we attempted to construct a prognostic model for patients with gastric cancer, as illustrated by the specific research route shown in the graphical abstract.

## 2 METHODS

### 2.1 The University of Alabama at Birmingham (UALCAN) and The Cancer Genome Atlas (TCGA) Databases

RNA transcriptional and protein expression profiles of the MMPs in gastric cancer and healthy gastric cells were collected using the UALCAN websites. To assess the mRNA levels of the MMPs, we chose UALCAN, a dynamic website for TCGA-STAD mRNA level data analysis [24-27]. After analyzing the TCGA database of UALCAN, differential expression levels of MMPs mRNA were examined in the gastric carcinoma group compared to the healthy individuals’ group. The gene expressions were also analyzed based on individual cancer stages.

### 2.2 Human Protein Atlas (HPA)

Nearly 20 different forms of cancer are covered by the HPA (https://www.proteinatlas.org), which provides details on how proteins are expressed in healthy tissues, cells, and cancers. In the current research work, proteins’ immunohistochemistry images made by the MMP families were obtained from the website to compare normal human gastric tissues to gastric cancer tissues [28].

### 2.3 Kaplan–Meier Plotter

To analyze the overall survival of a given gene, genetic information and clinical data were simultaneously integrated using the Kaplan–Meier plotter (http://kmplot.com/analysis/) [29, 30]. To investigate the Overall Survival (OS) prognostic value of different MMP family members in gastric cancer, the gastric cancer patients were divided into different sets based on an auto-select best cutoff.

### 2.4 cBioPortal

cBioPortal (http://www.cbioportal.org) is a platform that visualizes and analyzes large-scale tumor gene information from more than 100 cancer studies in the TCGA pipeline [31, 32]. We used the partial online database of TCGA-STAD. In gastric cancer, the gene characteristics of the MMPs family genes with mutations, structural variants, potential copy number alterations from GISTIC, and mRNA levels *z*-scores compared to diploid samples were investigated. OncoPrint was used to show the important alteration frequency of genes. A KM plot was made to display the connection between MMP mutations and survival time, and a log-rank test was conducted to find the significance of the difference between the survival curves.

### 2.5 Gene Expression Profiling Interactive Analysis (GEPIA)

GEPIA provides a range of analyses, including dimension reduction, Overall Survival (OS) analysis, similar expression detection, cancer/healthy differential analysis, and correlation analysis [33]. In this study, we selected the top 100 co-expressed mRNAs from the MMP gene group using the GEPIA 2 platform (http://gepia2.cancer-pku.cn) (Tables **S1** and **S2**).

### 2.6 Gene Ontology (GO) and Kyoto Encyclopedia of Genes and Genomes (KEGG) Analysis

The lists of genes produced from GEPIA can be functionally interpreted using the bioinformatics capabilities provided by the Database for Annotation, Visualization, and Integrated Discovery (DAVID) (https://david.ncifcrf.gov/summary.jsp) [34, 35]. To determine the functional roles of the top 100 co-expressed gene mutations, a GO enrichment analysis, including Biological Processes (BP), Cellular Components (CC), and Molecular Functions (MF), was performed using DAVID. The 100 co-expressed genes linked to the MMP family mutations and the pathways associated with MMPs were defined by performing KEGG analysis in DAVID. In this study, KEGG pathways and GO concepts were enriched using R 4.1.3 software to determine the significance of the different MMP family members and their co-expressed genes. We calculated the “GeneRatio” for the different GO and KEGG pathways and ranked them from top to bottom. The top 10 candidates were chosen and presented.

### 2.7 Search Tool for the Retrieval of Interaction Genes/Proteins (STRING)

Protein-Protein Interactions (PPIs) network functions as regulatory nodes in many cell-signaling networks associated with cancer’s “hallmarks”. Many PPIs that are closely linked to cell signaling and cell survival have been identified and validated as cancer biomarkers. STRING is a website for known and anticipated PPI. In this study, we used the “multiple proteins” channel to search for proteins that interact with the MMP family; the minimum required interaction score was set at 0.4, and a maximum of 10 interactors were displayed in the first shell [36].

### 2.8 Development of the Nomogram

Continuous variables were analyzed using the t-test or Mann-Whitney U test, while categorical variables were analyzed using the χ2 test or Fisher’s exact test. We used univariate analysis to analyze MMPs’ expressions. A multivariate Cox regression analysis was performed for the outcomes. We selected clinical factors and MMPs with p < 0.05 to develop the nomogram. Each variable in the nomogram had a corresponding weighted score and the sum of the weighted scores was associated with OS. The 1, 3, and 5-year calibration curves of the nomogram were plotted.

### 2.9 Ethics Statement

All the data were gathered from internet databases; hence, no ethics approval was required.

### 2.10 Statistical Methods

Using R 4.1.3 software, bioinformatic statistical analyses were performed (Library ggplot2, survival, plyr, riskRegression, rms, MASS, leaps, glmnet, ggDCA, and survivalROC). Continuous variables were analyzed using the t-test or Mann-Whitney U test, while categorical variables were analyzed using the χ2 test or Fisher’s exact test. Survival rates were calculated using the Kaplan-Meier method and compared using the log-rank test. Statistical significance was set at p < 0.05. The predictive significance of MMPs’ mRNA levels was investigated using univariate and multivariate Cox regression analyses. In the univariate and multivariate Cox regression analyses, we selected factors with p < 0.05. Each variable in the nomogram had a corresponding weighted score and the sum of the weighted scores was associated with OS.

## 3 RESULTS

### 3.1 The Expression Levels of MMPs mRNA and Protein in Gastric Carcinoma

**Fig. (1):**
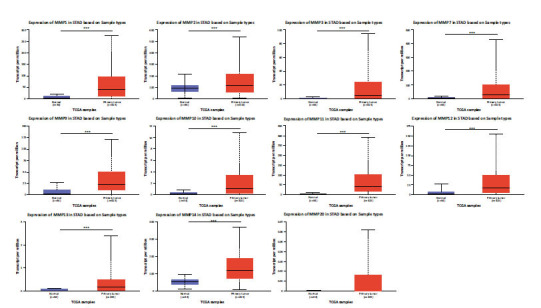
mRNA expression of MMPs in gastric cancer tissues and adjacent normal gastric tissues (UALCAN). mRNA expressions of MMP1/2/3/7/9/10/11/12/13/14 were found to be significantly elevated in gastric cancer tissues compared to normal samples, while no statistical differences in MMP20 mRNA expressions were observed between cancer tissues and normal tissues. ***, p < 0.001; **, p < 0.01; *, p < 0.05.

**Fig. (2):**
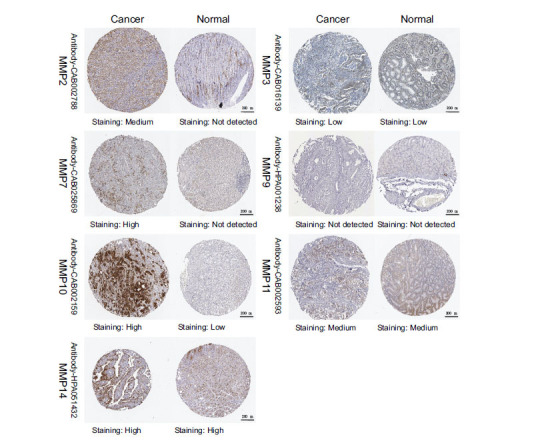
Representative immunohistochemistry images of MMPs in gastric cancer tissues and normal tissues (Human Protein Atlas).

Fig. **1** shows that the expression levels of MMP1–3, MMP7, and MMP9–14 were significantly higher in the gastric carcinoma samples compared to the healthy samples. However, MMP20 did not show a statistically significant difference between the normal and cancer tissues. Furthermore, using the HPA program, we attempted to determine the protein levels of MMPs in gastric cancer. As shown in Fig. **2**, MMP2 and MMP7 protein expression levels were not observed in the healthy samples, but they were present in the gastric cancer tissues at medium and high levels. However, outcomes were not statistically significant due to the small sample size. No expression of MMP2, MMP7, and MMP10 was observed in normal tissue samples (n=2). Of 12 gastric carcinomas (MMP2), in 2 tissues, the MMP2 levels were medium. While of 8 gastric carcinomas (MMP7), in 1 tissue, MMP7 was at a high level. In the case of the other 12 gastric carcinomas (MMP10), in 6 tissues, MMP10 was at medium/high level. The MMP1/3/9/10/11/14 protein levels were identical in both groups of tissues.

### 3.2 Relationship Between Gene Expression Levels of Various MMPs and Gastric Carcinoma Stage

**Fig. (3):**
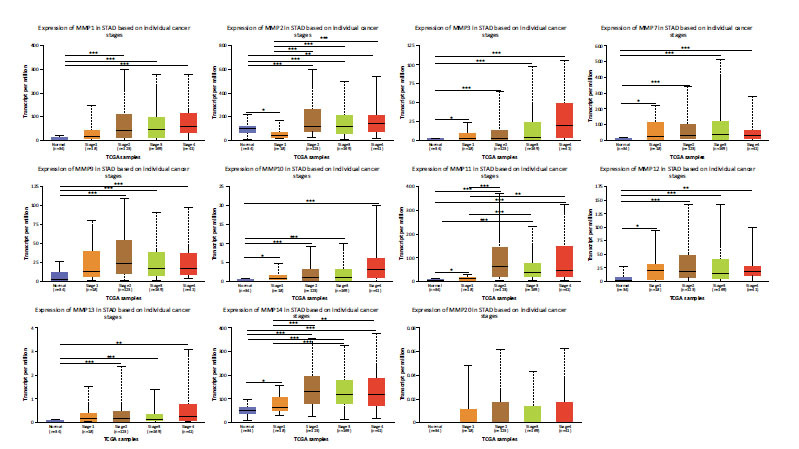
Correlation between MMP family expression and tumor stage in gastric cancer patients (UALCAN). The mRNA expressions of MMP 2/11/14 were significantly related to patients’ individual cancer stages, while mRNA expressions of MMP1/3/7/9/10/12/13/20 were not associated with patients' individual cancer stages. ***, p < 0.001; **, p < 0.01; *, p < 0.05.

The connection between the mRNA transcriptional levels of various MMPs and the distinct tumor stages of patients with gastric carcinoma was investigated using the UALCAN database. As shown in Fig. **3**, the MMP2/11/14 groups varied appreciably, and compared to stage 1, tumor tissue expression was higher in stages 2, 3, and 4.

### 3.3 Correlation Between the MMP Members and Survival Time of Gastric Cancer Group

**Fig. (4):**
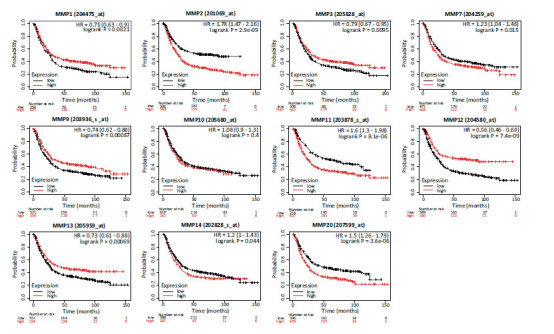
Prognostic features of mRNA expression of distinct MMP family members in gastric cancer patients (Kaplan–Meier plotter). The OS survival curves comparing patients with high (red) and low (black) MMP family members' expression in gastric cancer were plotted using the Kaplan–Meier plotter database at the threshold of the p-value of <0.05. Higher mRNA expressions of MMP2/7/11/14/20 were significantly associated with shorter Overall Survival (OS).

The Kaplan-Meier plotter was used to examine the survival results of MMPs in patients with gastric carcinoma. As observed in KM analysis, a total of 876 gastric cancer patients exhibited survival. Fig. **4** illustrates the association between gastric cancer patient prognosis and MMP expression levels. According to the log-rank test and Kaplan-Meier curve analysis, patients with gastric cancer who had elevated MMP2/7/11/14/20 expression levels had significantly reduced OS. In contrast, patients with upregulated MMP1/3/9/12/13 expressions had a significantly prolonged OS.

### 3.4 Genetic Alterations of MMP Gene Members in Patients with Gastric Cancer

**Fig. (5):**
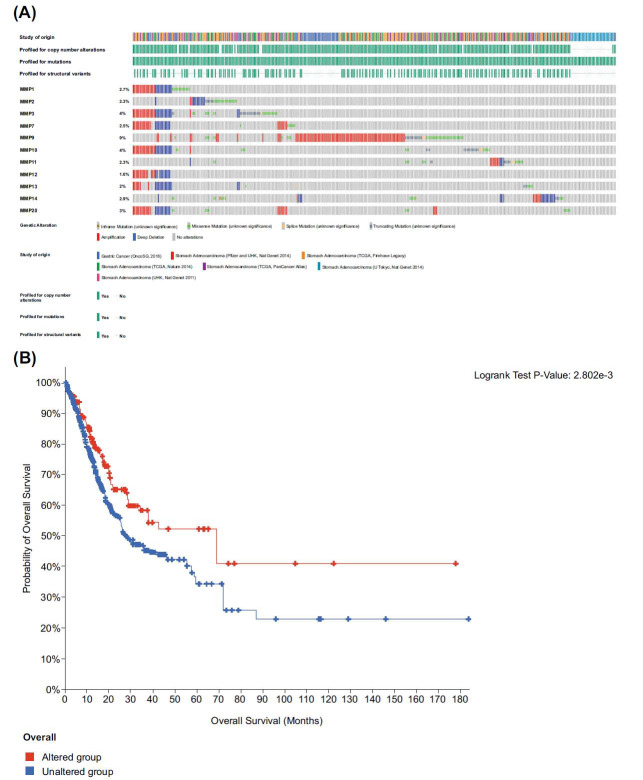
Genetic mutations in MMPs and their association with OS of gastric cancer patients (cBioPortal). (**A**), MMP9/3/10 ranked the highest three genes of genetic alterations, and their mutation rates were 9%, 4%, and 4%, respectively. (**B**), Kaplan–Meier plots comparing OS in cases with/without MMP genetic alterations.

cBioPortal was used to identify the gene mutations in MMP family members in the gastric cancer samples, and the results indicated that 315 of the 1512 patients with gastric cancer (21%) had mutations in the MMP family (Fig. **5A**). Individual members of the MMP family had genetic alteration percentages in gastric cancer, ranging from 1.6% to 9% (MMP1, 2.7%; MMP2, 2.3%; MMP3, 4%; MMP7, 2.5%; MMP9, 9%; MMP10, 4%; MMP11, 2.3%; MMP12, 1.6%; MMP13, 2%; MMP14, 2.9%; and MMP20, 3%). Additionally, the Kaplan–Meier curve and log-rank evaluation showed a prolonged OS in patients with gastric cancer as associated with genetic alterations in the MMPs (Fig. **5B**; p < 0.001). These findings indicated that the prognosis of patients with gastric cancer may be significantly affected by the genetic alterations in MMPs.

### 3.5 Investigation of MMPs’ Biological Functions and Signaling Pathways

**Fig. (6):**
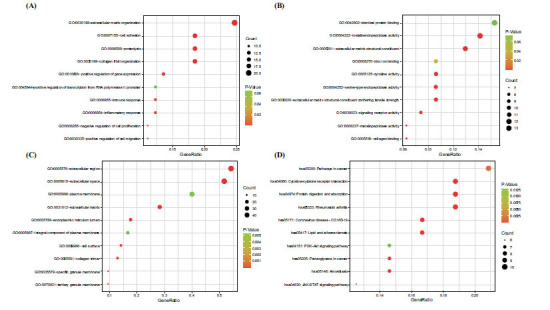
Pathway enrichment results. The best 10 pathways were enriched in the biological process, molecular function, cellular component, and KEGG pathway for MMPs genes. (**A**) BP, (**B**) MF, (**C**) CC, and (**D**) KEGG pathways.

**Fig. (7):**
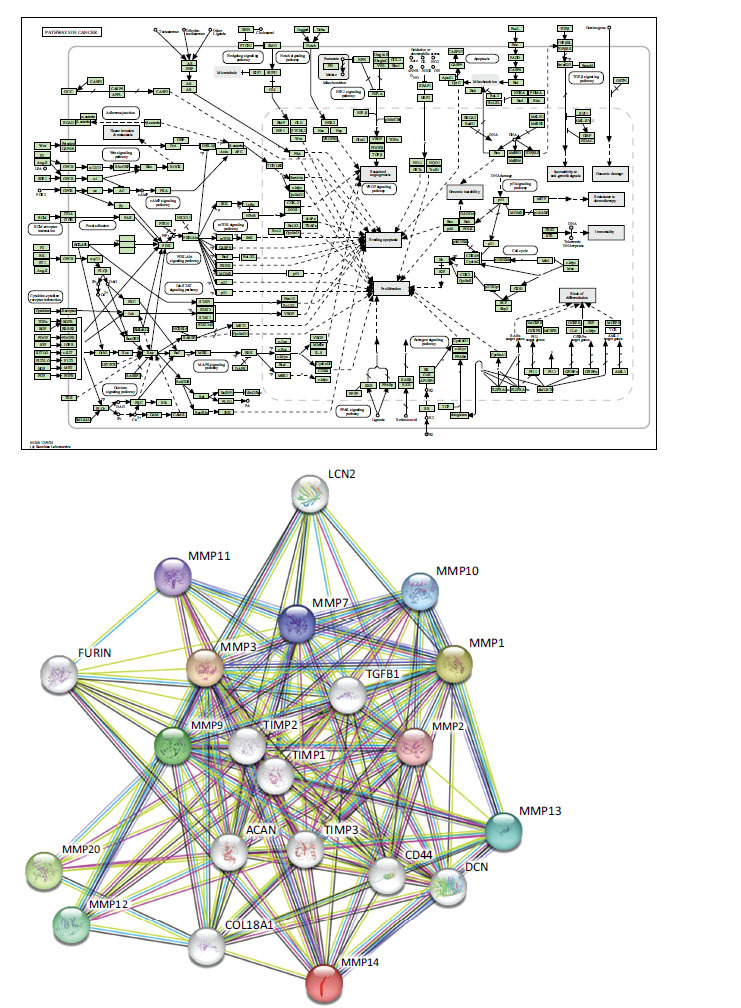
Top pathway in KEGG and PPI of MMPs. (**A**), “Pathway in cancer” in KEGG. (**B**), Protein–protein Interaction (PPI) network. The nodes represent proteins and the edges indicate the interaction of proteins.

The GO and KEGG analyses enriched pathways are presented in Figs. **6A-D**. Each top row of the bubble chart represents the pathway with the highest GeneRatio (R 4.1.3). The analysis of GO BP revealed MMPs to mostly be in association with positive regulation by identical protein binding, metalloendopeptidase activity, and extracellular matrix structure (Fig. **6A**). The MF analysis revealed that these differentially expressed proteins functioned mainly for extracellular matrix organization, cell adhesion, and extracellular matrix structural constituents (Fig. **6B**). The CC analysis predicted cellular components regulated by these differentially expressed proteins, including extracellular region, extracellular space, and plasma membrane (Fig. **6C**). KEGG pathway analysis could define the molecular pathways in which MMPs and other interacted genes were involved. The top 10 pathways were shown by the KEGG results that were related to the functions of MMPs in gastric cancer (Fig. **6D**). Fig. **7A** shows the top KEGG enrichment pathway item “pathway in cancer”. To create a PPI network with the MMPs, we searched the STRING website for members connected with MMPs (Fig. **7B**). No more than 10 clusters were chosen to appear in the first shell. Text mining, experiments, databases, co-expression, neighborhood, gene fusion, and co-occurrence were included as sources of active interaction. The network was constructed using 21 proteins, including MMP1/2/3/7/9/ 10/11/12/13/14/20, LCN2, FURIN, TGFB1, TIMP1, TIMP2, TIMP3, ACAN, CD44, DCN, and COL18A1.

### 3.6 Development of the Nomogram

**Fig. (8):**
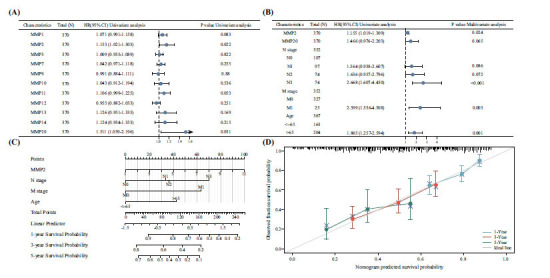
Development and performance of the nomogram. (**A-B**), Cox regression analysis of MMPs and clinical factors in gastric cancer patients. (**C**), Nomogram based on prognostic factors. (**D**), 1-, 3-, 5-year calibration curves.

Continuous variables were analyzed using the t-test or Mann-Whitney U test, while categorical variables were analyzed using the χ2 test or Fisher’s exact test. Survival rates were calculated using the Kaplan-Meier method and compared using the log-rank test. Statistical significance was set at p < 0.05. Univariate Cox regression showed MMP2 [HR: 1.153 (1.021-1.303)] and MMP20 [HR: 1.511 (01.039-2.196)] as risk factors for gastric cancer patients (Fig. **8A**). Four independent risk factors (p < 0.05) were identified by multivariate analysis: MMP2, N stage, M stage, and age (Fig. **8B**). We constructed a nomogram model according to the selected variables using the multivariate analysis model, wherein each patient's total weighted score was calculated, and the corresponding score was obtained (Fig. **8C**). The C-index was obtained as 0.651 (0.625-0.677). Fig. **8D** shows the 1-, 3-, 5-year survival calibration curves.

## DISCUSSION

There has been an increase in research over the past decade that not only explains how MMP family members contribute to tumorigenesis and proliferation, but also how they might be targeted for the treatment of various cancers [12, 37-42]. Mounting evidence suggests that the MMP family of genes is frequently aberrantly expressed in cancers [39, 43-45]. In this study, we have presented a comprehensive comparison of the mRNA levels of MMP genes in gastric carcinoma and healthy samples. The findings have suggested that the overwhelming majority of the MMP family is overexpressed in cancer tissues, thus demonstrating potential diagnostic value in gastric cancer. Notably, MMP2, MMP7, and MMP10 showed potential differences in immunohistochemical assays. However, MMP10 overexpression did not show a difference in OS in the database. This may be because its overexpression is a feedback-induced elevation in the tumor by other genes, or its effect on the OS is controlled by the function of other MMP family members. Based on this presentation of MMP10, we do not consider that MMP10 is a suitable predictive target. The results also showed that MMP2 and MMP7 may be the better diagnostic biomarkers. Several studies have shown MMP2 to be currently the most potent therapeutic target in the MMP family, and a large amount of research has been conducted to target MMP2 [46-49]. In animal models, generic MMP-2 inhibitors have been shown to prevent tumor dissemination and metastasis. In addition, as the smallest member of the MMP family, MMP-7 is a crucial factor in gastric cancer progression and is involved in its occurrence and development [50-53]; therefore, it might be an independent survival biomarker for gastric cancer [54-56]. Therefore, these two molecules are important for future research. At the same time, we found that MMP1/3/7/9/12/13/20 expression does not correlate with gastric cancer stage. The reason the transcriptional levels of these MMPs in the gastric cancer tissues did not differ considerably may be attributed to the limited sample size.

According to the data, the poor patient prognosis was related to the over-expression of MMP2/7/11/14/20 and the low expression of MMP1/3/9/12/13. However, it is crucial to recognize that the molecular numbers of the MMP family are large, the degree of expression correlates with each other, and further analysis is needed to identify critical molecules for gastric cancer survival. At the same time, the functions of many newer members of the MMP genes have been revealed; in the field of cancer, the members and interactions of the MMP family are becoming increasingly complex. Analyzing the interplay between these MMPs and developing multiple targeted inhibitors could potentially improve patient survival [42, 57].

A low alteration rate (21%) in the MMPs was observed in the gastric carcinoma tissues, and genetic alterations in the MMPs were related to a good OS. This is because mutations in the MMP family may lead to functional upregulation of MMPs. The mutation rates were highest for MMP9 (9%) and MMP3 (4%) among the MMPs. Upregulation of function caused by MMP3/9 mutations played a significant role in prognosis, with high function of MMP3/9 associated with prolonged overall survival in gastric cancer patients. As one of the most intricate types of MMPs, MMP9 may break down ECM components and play a significant role in the pathophysiological processes [58-61]. According to some studies, controlling MMP-9 is a crucial therapeutic strategy for treating many diseases, including cancer [62, 63]. Thus, the bilateral function of MMP-9 in cancer warrants further investigation.

Additionally, the MMPs and their 100 co-expressed genes completed the functional enrichment analysis, and the findings confirmed the involvement of MMPs in various GO processes, including “identical protein binding”, “metalloendopeptidase activity”, and “extracellular matrix structure”. There are studies that show that Epithelial-mesenchymal Transition (EMT) plays an important role in the metastasis of gastric cancer. EMT was able to render gastric cancer cells more detachable, with simultaneous distant metastasis. The above three GO processes are closely related to EMT. MMPs may, therefore, promote metastasis of gastric cancer [64-66]. In addition, the MMPs were also found to be involved in a variety of KEGG pathways, such as “pathways in cancer”, “cytokine–cytokine receptor interaction”, “protein digestion and absorption”, “rheumatoid arthritis”, and “Coronavirus Disease (COVID-19)”. In this study, we discovered that the MMP family primarily affects not only tumor-associated signaling pathways, but also normal tissue physiology. This could be the main reason why many of the inhibitors are not safe enough for clinical use [41, 67, 68]. In this study, we discovered that the MMP family primarily affects not only tumor-associated signaling pathways, but also normal tissue physiology. In addition, protein digestion and absorption are also an important part of the tumor's normal work. From “rheumatoid arthritis” to “Coronavirus Disease (COVID-19)” pathways, MMPs are strongly associated with unrestricted extracellular matrix proliferation [69]. This may explain, in part, the predisposition of gastric cancer to peritoneal metastases.

Our interaction analysis revealed that MMP7 has extensive connections with nearly all other MMP family members. Additionally, its interaction with key molecules, such as Furin, TGFB1, and CD44, underscores its influence on crucial cancer pathways [44, 70-72]. Furin is the first discovered proprotein convertase member. Since many of these protein precursors are involved in initiating and maintaining the hallmarks of cancer, Furin has been proposed as a potential target for treating several human cancers. TGFB1 acts as a tumor promoter of colorectal cancer and plays an important role in EMT. The TGFB maturation step requires Furin activity. CD44 modulates cellular adhesion, migration, and growth, which also plays a pivotal role in driving cancer resistance and EMT [73-76]. Therefore, the effective management of the MMP family’s expressions can prevent gastric cancer progression via EMT pathways.

Finally, we sought to construct a prognostic model of MMPs in combination with clinical factors. MMP2 expression emerged as an independent risk factor for mortality. The nomogram constructed by MMP2, N stage, M stage, and age has exhibited great predictive performance. This result further suggested MMP2 to be an important potential biomarker [46-48]. In the future, the MMPs family can play an important role in diagnosis and treatment. Nowadays, target therapy is gradually improving, and both ADCs and small-molecule inhibitors can target important tumor genes with good efficacy [77-79]. Currently, drugs targeting MMP7 have emerged. MMP2 may be another new target selection of the MMP family.

## CONCLUSION

Most members of the MMPs family are highly expressed in gastric cancer tissue, and differential expressions of MMPs can significantly influence patient prognosis. At the same time, mutations in the family of MMPs generally improve patient outcomes. In addition, an analysis of the functions and pathways of the MMPs family suggested that the MMPs family influences the development of various aspects of gastric cancer, and can prove to be a potential biomarker and therapeutic target. Finally, we constructed a clinical prediction model including MMP2 that could accurately predict survival in gastric cancer patients.

## Data Availability

All data are available in the public repositories (TCGA, Human Protein Atlas, STRING, GO, KEGG). The datasets generated during the current study are available in the following links: 1. Figure **1**, Figure **3**: ualcan.path.uab.edu 2. Figure **2**: www.proteinatlas.org 3. Figure **4**: www.kmplot.com 4. Figure **5**: www.cbioportal.org 5. Figure **6**: david.ncifcrf.gov/summary.jsp; gepia.cancer-pku.cn/ 6. Figure **7**: www.genome.jp; cn.string-db.org 7. Figure **8**: portal.gdc.cancer.gov.

## LIST OF ABBREVIATIONS

MMPs: Matrix Metalloproteinases
UALCAN: The University of Alabama at Birmingham
HPA: Human Protein Atlas
GEPIA: Gene Expression Profiling Interactive Analysis
STRING: Search Tool for the Retrieval of Interaction Gene/Proteins
DAVID: Database for Annotation, Visualization, and Integrated Discovery
ECM: Extracellular Matrix
TCGA: The Cancer Genome Atlas
OS: Overall Survival
GO: Gene Ontology
KEGG: Kyoto Encyclopedia of Genes and Genomes
BP: Biological Processes
CC: Cellular Components
MF: Molecular Functions
PPIs: Protein-Protein Interactions
