# Supplementary material for: A Comprehensive Analysis of the Clinical Significance and Underlying Oncogenic Roles of Specific MMPs in Gastric Carcinoma Reveals their Potential Roles in Prognosis and Therapy
Source: Curr Mol Med. 2025 Jan 3;25(11):1427–40. doi: 10.2174/0115665240309837241204184939 (PMC13266698; doi:10.2174/0115665240309837241204184939)
Supplement: Supplementary file 1 [file CMM-25-11-1427_SD1.pdf]

A comprehensive analysis of the clinical significance and underlying oncogenic roles of specific MMPs in gastric carcinoma reveals their potential roles for prognosis and therapy

Short title: MMPs in gastric cancer

Shiyang Jin[1#], Jing Wang2[#], Kuan Wang[1*]

1. Department of Gastrointestinal Surgery. Harbin Medical University Cancer Hospital, Harbin, China
2. Department of Breast Cancer Surgery. Harbin Medical University Cancer Hospital, Harbin, China

[#]Co-first authors：

Shiyang Jin, 784224552@qq.com

Jing Wang, 931105187@qq.com

[*]Corresponding author:

Kuan Wang

Department of Gastrointestinal Surgery.

Harbin Medical University Cancer Hospital.

No. 150, Haping Road, Nangang District, Harbin 150040, Heilongjiang, China.

0696@hrbmu.edu.cn.

+86-0451□86298071.

Supplementary Material

Table S1: 100 co-expressed gene of MMPs

Table S2：characteristics of the patients

Table S1：100 co-expressed gene of MMPs

Gene Symbol	Gene ID	PCC
LOXL2	ENSG00000134013.15	0.73
PRR16	ENSG00000184838.14	0.73
PDPN	ENSG00000162493.16	0.7
COL12A1	ENSG00000111799.20	0.68
WNT5A	ENSG00000114251.13	0.67
COL5A2	ENSG00000204262.11	0.67
TREM1	ENSG00000124731.12	0.65
MMP3	ENSG00000149968.11	0.64
COL7A1	ENSG00000114270.15	0.63
SLC11A1	ENSG00000018280.16	0.63
INHBA	ENSG00000122641.9	0.63
NID2	ENSG00000087303.16	0.63
ADAMTS2	ENSG00000087116.13	0.63
MMP14	ENSG00000157227.12	0.62
CTB-114C7.4	ENSG00000250274.1	0.61
COL5A3	ENSG00000080573.6	0.61
CLEC5A	ENSG00000258227.6	0.61
ADAMTS12	ENSG00000151388.10	0.6
SULF1	ENSG00000137573.13	0.6
TMEM158	ENSG00000249992.1	0.59

ADAM12	ENSG00000148848.14	0.59
UCN2	ENSG00000145040.3	0.59
FPR2	ENSG00000171049.8	0.58
CXCL8	ENSG00000169429.10	0.58
CTD-2334D19.1	ENSG00000248927.1	0.58
CCL3	ENSG00000277632.1	0.58
TLR2	ENSG00000137462.6	0.58
FAM196B	ENSG00000204767.3	0.58
SOD2	ENSG00000112096.16	0.58
CLEC4D	ENSG00000166527.7	0.57
GPR84	ENSG00000139572.3	0.57
AQP9	ENSG00000103569.9	0.57
HIF1A	ENSG00000100644.16	0.57
LAMC3	ENSG00000050555.17	0.56
CDH13	ENSG00000140945.15	0.56
FAP	ENSG00000078098.13	0.56
MMP19	ENSG00000123342.15	0.56
CLEC4E	ENSG00000166523.7	0.56
CTB-61M7.2	ENSG00000268734.1	0.56
IL6	ENSG00000136244.11	0.56
SPATA20P1	ENSG00000231123.1	0.56
WISP1	ENSG00000104415.13	0.55

FCAR	ENSG00000186431.18	0.55
NLRP3	ENSG00000162711.16	0.55
WNT5A-AS1	ENSG00000244586.1	0.55
WNT2	ENSG00000105989.8	0.55
PODNL1	ENSG00000132000.11	0.55
PALM2-AKAP2	ENSG00000157654.17	0.55
MMP2	ENSG00000087245.12	0.54
AC004538.3	ENSG00000230333.6	0.54
C1QTNF6	ENSG00000133466.13	0.54
BMP1	ENSG00000168487.17	0.54
DCBLD1	ENSG00000164465.18	0.54
IL11	ENSG00000095752.6	0.54
PI15	ENSG00000137558.7	0.54
CD300E	ENSG00000186407.6	0.54
GPR176	ENSG00000166073.8	0.54
ADAMTS7	ENSG00000136378.14	0.54
ANTXR1	ENSG00000169604.19	0.53
BCL2A1	ENSG00000140379.7	0.53
LILRB3	ENSG00000204577.11	0.53
SGIP1	ENSG00000118473.21	0.53
CHI3L1	ENSG00000133048.12	0.53
ADGRG3	ENSG00000182885.16	0.53

MMP1	ENSG00000196611.4	0.53
FAM132B	ENSG00000178752.15	0.52
COL4A1	ENSG00000187498.14	0.52
COL1A2	ENSG00000164692.17	0.52
CSF2	ENSG00000164400.5	0.52
PXDN	ENSG00000130508.10	0.52
SPATA13	ENSG00000228741.2	0.52
ADAMTS4	ENSG00000158859.9	0.51
HS3ST3A1	ENSG00000153976.2	0.51
PFKFB3	ENSG00000170525.18	0.51
ACAN	ENSG00000157766.15	0.51
IL13RA2	ENSG00000123496.7	0.51
MMP10	ENSG00000166670.9	0.51
FCGR2A	ENSG00000143226.13	0.51
ITGAX	ENSG00000140678.16	0.5
AP001434.2	ENSG00000226012.1	0.5
RGS16	ENSG00000143333.6	0.5
COL6A3	ENSG00000163359.15	0.5
GPR4	ENSG00000177464.4	0.5
RP11-264E20.1	ENSG00000255465.3	0.5
PLAUR	ENSG00000011422.11	0.5
HCK	ENSG00000101336.12	0.5

XIRP1	ENSG00000168334.8	0.5
IL24	ENSG00000162892.15	0.5
GK-IT1	ENSG00000229331.1	0.5
MCEMP1	ENSG00000183019.7	0.49
POSTN	ENSG00000133110.14	0.49
COL5A1	ENSG00000130635.15	0.49
PDGFB	ENSG00000100311.16	0.49
BMP8A	ENSG00000183682.7	0.49
FAM65C	ENSG00000042062.11	0.49
COL13A1	ENSG00000197467.13	0.49
P3H1	ENSG00000117385.15	0.49
LOX	ENSG00000113083.12	0.49
CTSK	ENSG00000143387.12	0.49
ZNF469	ENSG00000225614.2	0.48

Table S2: **characteristics of the patients**

Characteristics	Low expression of MMP2	High expression of MMP2	P value
n	173	162	
Age, n (%)			0.657

Table S2: **characteristics of the patients**

Characteristics	Low expression of MMP2	High expression of MMP2	P value
<= 65	80 (23.9%)	71 (21.2%)	
> 65	93 (27.8%)	91 (27.2%)	
Gender, n (%)			0.430
Male	106 (31.6%)	106 (31.6%)	
Female	67 (20%)	56 (16.7%)	
Pathologic T stage, n (%)			0.004
T1	16 (4.8%)	1 (0.3%)	
T2	35 (10.4%)	34 (10.1%)	
T3	79 (23.6%)	79 (23.6%)	
T4	43 (12.8%)	48 (14.3%)	
Pathologic N stage, n (%)			0.903
N0	55 (16.4%)	49 (14.6%)	
N1	48 (14.3%)	42 (12.5%)	
N2	37 (11%)	35 (10.4%)	

Table S2：**characteristics of the patients**

Characteristics	Low expression of MMP2	High expression of MMP2	P value
N3	33 (9.9%)	36 (10.7%)	
Pathologic M stage, n (%)			0.427
M0	162 (48.4%)	148 (44.2%)	
M1	11 (3.3%)	14 (4.2%)	
